# Multitask Quantum Study of the Curcumin-Based Complex Physicochemical and Biological Properties

**DOI:** 10.3390/ijms23052832

**Published:** 2022-03-04

**Authors:** Kaouther Baira, Ali Ounissi, Hafida Merouani, Manawwer Alam, Nadia Ouddai, Alessandro Erto, Krishna Kumar Yadav, Saiful Islam, Ji-Kwang Cheon, Byong-Hun Jeon, Yacine Benguerba

**Affiliations:** 1 Laboratoire de Chimie des Matériaux et des Vivants, Activité & Réactivité (LCMVAR), Université Batna 1, Batna 5000, Algeria; baira_kaouther@yahoo.fr (K.B.); aliounissi2015@gmail.com (A.O.); merouani_hafida@yahoo.fr (H.M.); ouddai_nadia@yahoo.fr (N.O.); 2 Department of Process Engineering, Faculty of Technology, University Ferhat ABBAS Sétif 1, Sétif 19000, Algeria; 3 Department of Chemistry, College of Science, Kind Saud University, P.O. Box 2455, Riyadh 11451, Saudi Arabia; maalam@ksu.edu.sa; 4 Dipartimento di Ingegneria Chimica, dei Materiali e della Produzione Industriale, Università degli Studi di Napoli Federico II, Piazzale Vincenzo Tecchio 80, 80125 Naples, Italy; aleserto@unina.it; 5 Faculty of Science and Technology, Madhyanchal Professional University, Ratibad, Bhopal 462044, India; envirokrishna@gmail.com; 6 Department of Geotechnics & Transportation, School of Civil Engineering, Faculty of Engineering, Universiti Teknologi Malaysia, Johor Bahru 81310, Malaysia; saiful.islam.iitr@gmail.com; 7 Department of Earth Resources and Environmental Engineering, Hanyang University, Seoul 04763, Korea; jkcheon@hanyang.ac.kr

**Keywords:** curcumin, metal complex, ETS-NOCV, QTAIM, TDDFT, COSMO-RS

## Abstract

Density functional theory (DFT), time-dependent density functional theory (TDDFT), quantum theory of atoms in molecules (QTAIM), and extended transition state natural orbitals for chemical valence (ETS-NOCV) have all been used to investigate the physicochemical and biological properties of curcumin and three complexes, i.e., Cur-M (M = Ni, Cu, and Mg). Based on DFT calculations, the enolic form (Cur-Enol) is more stable than the anti-diketone form (Cur-Anti diketone) favored for complexation. This enolic form stability was explained by the presence of three intramolecular hydrogen bonds according to the QTAIM analysis. Furthermore, the ETS-NOCV technique revealed that the enolic form had more significant antioxidant activity compared with the anti-diketone form. The calculations from the COnductor-like Screening MOdel for Realistic Solvents (COSMO-RS) showed that the dimethyl sulfoxide (DMSO) solvent could dissolve all the curcumin tautomers Cur-Enol, Cur-Anti-diketone and Cur-Cu, Cur-Mg, and Cur-Ni complexes in contrast to benzene, acetone, octanol, ethanol, methanol, and water. Furthermore, except for Cur-Mg, which had a relatively low solubility (14 g/L), all complexes were insoluble in water. Cur-Anti-diketone was considerably more soluble than Cur-Enol in the examined solvents.

## 1. Introduction

Curcumin, a major active component of Turmeric, has long been used as a spice, and it possess a wide range of biological activities, including antibacterial and antifungal [1], antioxidant, anticancer [2,3], antimicrobial [4], and inflammatory properties [5]. Curcumin has piqued the interest of many academics since then, and numerous papers have been published on the subject [6,7,8]. It was first discovered by Vogel and Pelletier as a powder “yellow coloring matter” from rhizomes of *C. longa* (Zingiberaceae family) [9], and characterized and first synthesized by Milobedeska, Lampe et al. [10,11]. 

The chemical structure was investigated by Heger et al. [12] as [(1*E*, 6*E*)-1,7-bis(4-hydroxy-3-methoxy-phenyl)-1,6-heptadiene-3,5-dione]. Curcumin has a melting point of 183 °C, molecular formula of C_21_H_20_O_6_, and molecular weight of 368.37 g/mol. Ketone and enol are the two tautomeric forms of curcumin that impact its complexation process, physicochemical, and biological characteristics [13].

Polyphenols have gained attention essentially due to their antioxidant capabilities. They can trap free radicals and prevent lipid peroxidation by scavenging free radicals. They can also capture metal ions due to their chelating capabilities. Nevertheless, curcumin complexes remain little explored despite their promising properties particularly for Alzheimer’s disease [14]. In general, the experimental identification of therapeutic characteristics of specific natural compounds requires a prior deep understanding, specifically for computational investigations. Examples include the solubility in water and other liquids of interest, especially when dealing with chemicals intended for human consumption. The insoluble nature of curcumin, for example, makes it challenging to utilize in medicinal applications.

The complexation of a chemical with other metals can drastically alter certain molecular features, resulting in new compounds with distinct properties that are potentially suitable for desired purposes. This is the case of curcumin and its complexes. Hence, the main purpose of this investigation is the study of the complexation behavior and the antioxidant properties of curcumin–metal complexes based on Nickel, Copper, and Magnesium transition metals by density functional theory (DFT) techniques, i.e., time-dependent density functional theory (TDDFT) to simulate the absorption and emission spectra; quantum theory of atoms in molecules (QTAIM) to simultaneously explore the electron density and hydrogen bond interactions; extended transition state natural orbitals for chemical valence (ETS-NOCV) to evaluate the antioxidant activity; and COSMO-RS to calculate and analyze the solubility of curcumin and molecular interactions in various solvents. The results will be helpful to the experimentalists to synthesize curcumin complexes with more reliable properties.

## 2. Computational Methods

As part of the DFT (density functional theory) level computations, the geometric optimization calculations were conducted using Gaussian09 [15] and the functional B3LYP (Becke three-parameter Lee–Yong–Parr) [16]. All the atoms were based on the 6-311G (2d, 2p) basis. The polarizable continuum model (PCM) introduced the DMSO (dimethyl sulfoxide) solvent effect. Casida’s method obtained characteristics and absorption spectrum of the excited states [17]. 

The theoretical study of curcumin-based transition metal compounds performed by Density Functional Theory (DFT) [18,19] using ADF software (Amsterdam Density Functional) [20] was only used for the single-point calculations, the functional GGA: PW91 (Generalized Gradient Approximation) [21], the Slater TZP (Triple zeta polarized) set of atomic bases [22], valence orbitals of all atoms (4s and 3d for Ni, Cu, and Mg; 2s and 2p for O and C; and 1s for H. Relativistic effects were taken into account at the scalar level using the regular approximation of order zero (ZORA) [23]. 

The integration parameter and the energy convergence criterion are: 6 and 10-6 a.u., respectively. Several DFT approaches can be used to describe the properties of chemical bonds; the quantum theory of atoms in molecules (QTAIM) is one of them. Hydrogen bonds were characterized using QTAIM [24], which was connected with investigating electron density’s topology [24,25,26]. Using the NOCV technique [27], the dissociation energy of the phenolic hydrogen atoms could be calculated, allowing the antioxidant activity of the molecule and its complexes to be measured. 

According to the relevant research [28,29], the ETS technique was included in the NOCV method [30,31]. Covalent bonds [32], intramolecular agnostic interactions [29,31], and intermolecular hydrogen bonds [33] may all be determined using the combined ETS-NOCV technique. The binding interactions were investigated using a Morokuma-type energy decomposition described by Ziegler and Rauk [34]. According to the stated approach, the total binding energy (Δ*E*) is defined as:

(1) $$\Delta E_{int}=\Delta E_{pauli}+\Delta E_{els}+\Delta E_{orb}$$

Δ*E_pauli_* indicates the repulsive four-electron interactions between the occupied orbitals, and Δ*E_els_* represents the classical electrostatic interaction between the molecule fragments in the complex in their final locations. Finally, the term Δ*E_orb_* stabilizes interactions between occupied and empty molecular orbitals of the two fragments. It should be emphasized that the last term Δ*E_orb_* contains the combination of occupied and vacant orbitals in the same fragment (polarization of internal fragments).

The strain density can be expressed as a sum of eigenvectors, $\psi_{-k},\;\psi_{+k}$ corresponding to the eigenvalues $-\nu_{k}$ and $+\nu_{k}$ with the same absolute value:

(2) $$\Delta \rho \left(\vec{r}\right)=\overset{M/2}{\underset{k=1}{\sum }}\nu_{k}\left[-\psi_{-k}^{2}\left(\vec{r}\right)+\psi_{+k}^{2}\left(\vec{r}\right)\right]=\overset{M/2}{\underset{k=1}{\sum }}\Delta \rho_{k}\left(\vec{r}\right)$$

where *M* denotes the total number of molecular orbitals on the fragments.

Specifically, all occupied and empty molecular orbitals of the two fragments are included in the summation. Its eigenvalue $\nu_{k}$ indicates the number of electrons that are moved from the anti-bonding orbitals, ψ−k. As a function of the eigenvalues of NOCV ($\nu_{i}$), the orbital interaction term (Δ*E_orb_*) is represented in the combined ETS-NOCV scheme as follows:

(3) $$\Delta E_{orb}=\overset{M/2}{\underset{k=1}{\sum }}\Delta E_{orb}^{k}=\overset{M/2}{\underset{k=1}{\sum }}\nu_{k}\left[T_{k,k}^{TS}-T_{-k,-k}^{TS}\right]$$

The diagonal elements of the Kohn–Sham matrix on NOCVs in the transition state (TS) are $T_{-k,-k}^{TS}$ and $T_{k,k}^{TS}$. 

Equations (2) and (3) show that $\Delta \rho_{k}$ can be visualized, and its energetic contributions to the overall bond energy are provided [26]. COSMO-RS is a completely predictive model for thermodynamic characteristics of fluids and solutions using statistical thermodynamics and quantum chemistry [35]. Polarization occurs when the charge density is high due to the solvent at the cavity surface. The total energy may be calculated by calculating the forces that result.

COSMO-RS combines statistical thermodynamics with quantum chemistry to provide an utterly predictive model for the thermodynamic characteristics of fluids and solutions. The charge density of the solvent at the cavity’s surface expresses its polarization effects. The resulting force was quantified to calculate the total energy. The COSMO-RS model is one of the most outstanding forecasters of solubility, particularly for tiny compounds, despite its lack of popularity [36].

## 3. Results and Discussion

### 3.1. The Antioxidant Property of Curcumin

The antioxidant property of curcumin depends fundamentally on its structure, including methoxylated phenols and the enol form of β-diketone. By Michael’s non-enzymatic addition or GST (Glutathione *S*-transferase) mediated process, curcumin’s core β-diketone component is converted from curcumin to glutathione. Curcumin has been found to be ten-times more effective than vitamin E as an antioxidant because of these particular characteristics [37].

**Figure 1:**
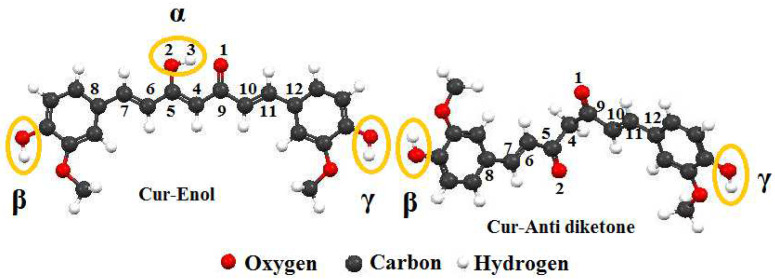
Optimized structure of Cur-Enol and Cur-Anti diketone at the B3LYP/6-311 G (2d, 2p) level of theory. (α, β, and γ are the active sites of both forms of curcumin).

**Figure 2:**
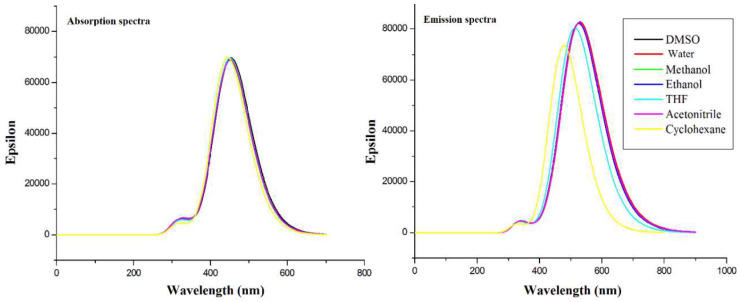
Absorption and emission spectra of the curcumin enol form (Cur-enol). (ε is the absorption intensity).

Curcumin was studied in two forms, Cur-Enol and Cur-Anti diketone. Both forms were neutral, and it was found that the Cur-Enol form had the best antioxidant properties (see Figure 1). The local antioxidant property was measured using ETS-NOCV and the Bond Dissociation Energy (BDE) (see Table 1 and Figure 2).

The Cur-Enol molecule has the best reducing characteristics (electron or hydrogen atom donor) due to the low dissociation energy value in phenol at the Position β and the low ionization potential (Table 1), which allows it to perform the antioxidant role. Our results agree with previous research findings based on experimental evaluations [38,39,40].

### 3.2. Absorption and Emission Wavelengths

Cur-enol form characterization has been the subject of research. The TD/B3LYP/6-311G (2d,2p) technique was used to theoretically compute absorption and emission wavelengths of curcumin in various solvents (DMSO, water, methanol, ethanol, THF, acetonitrile, and cyclohexane). Table 2 summarizes all the acquired findings, including the absorption/emission wavelength (λ_max_), oscillator strength f, and the Stokes shift (∆λ). Figure 2 shows the spectra of epsilon (ε) as a function of the wavelength.

The solvent DMSO produced the maximum absorption wavelength for curcumin enol (Table 2). The absorption wavelength is shorter in apolar solvents particularly cyclohexane, (λ_abs_ = 445.46 nm). The curcumin enol-form absorbs light in the following order: DMSO > ethanol > acetonitrile > water > THF > methanol > cyclohexane.

The results of Table 2 were further supported by Figure 2 demonstrates that the absorption spectra in all the solvents are almost identical. When comparing different solvent emission wavelengths λ_em_, water has the maximum value. In the enol-form of curcumin, the wavelength of λ_em_ emission decreases from cyclohexane to water (the most polar). In polar solvents, the oscillator force f, which reflects the transition probability, is more significant than in non-polar solvents. 

The order of the emission wavelengths in different media is given as: water > DMSO > acetonitrile > methanol > ethanol > THF > cyclohexane. The solvatochromic effect is weak but present in both absorption and emission events. The HOMO-LUMO transition of Cur-Enol in the investigated solvents dominates both the absorption and emission in the UV-vis range.

### 3.3. Curcumin Complexation

Curcumin is an efficient metal ion chelator, the diketone portion of curcumin can bind metals, such as copper, nickel, and magnesium. According to most scientific studies, the oxygen of the diketone part binds metal cations; whereas, other researchers suggested that the non-binding doublets of oxygen in the methoxy group might constitute chelation sites [41]. The chelation capacity of curcumin is particularly noteworthy since it can indirectly attach to proteins by utilizing metal or metalloprotein atoms. 

**Figure 3:**
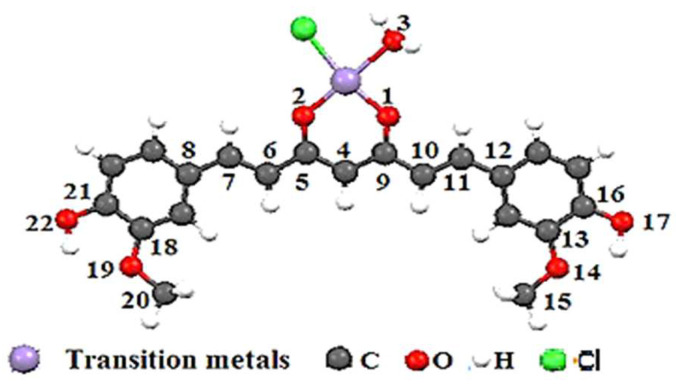
The studied structures of the Cur-M complexes (where M = Ni, Cu, and Mg) optimized at the PW91/TZP level of theory.

The geometry optimization was carried out by a “single point” calculation with PW91/TZP for curcumin complexes and B3LYP/6-311G (2p, 2d) for the curcumin ligand. The PW91/TZP was the most useful in reproducing the experimental data. The curcumin complex (neutral compounds) theoretical geometric characteristics (Figure 3 and Table 3) are similar to the literature results [42,43,44]. The spin states for the compounds Cur-Ni, Cur-Cu, and Cur-Mg are doublet, triplet, and singlet respectively.

**Figure 4:**
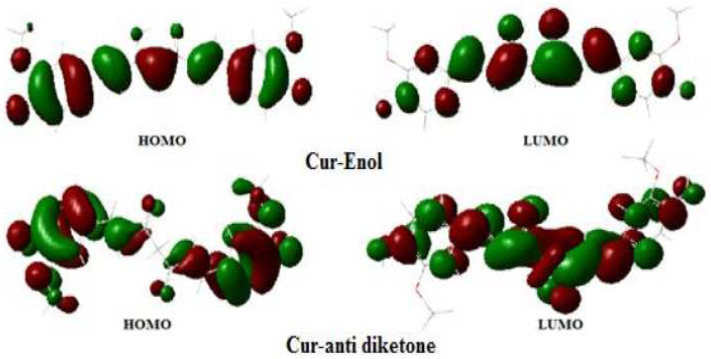
DFT contour plots of the HOMO and LUMO states of Cur-Enol and Cur-Anti-diketone (positive lobs are in green and negative lobs in blood-red); Isovalue = 0.02.

After complexation, the differences in binding lengths of specific molecule segments (C(4)–C(5)) and (C(4)–C(9)) (Figure 3) for both forms of curcumin allowed us to establish whether the complexation was produced using the enol or diketone form (Table 3). After complexing with the three metals, the bond lengths of Cur-Anti-diketone (C(4)–C(5)) and (C(4)–C(9)) decrease. On the other hand, the Cur-Enol complexation causes an increase in the C(4)–C(5) bond length and a decrease in the C(4)–C(9) bond length (Table 3). The nodal structure of the HOMO and LUMO orbitals of the two curcumins may explain this variation (Figure 4). Given that neither C(4), C(5) nor C(9) atoms in Cur-Anti diketone contribute to the HOMO orbitals, the LUMO structure may be the cause of the shift.

The reduced length of the two bonds is explained by the binding character of the C(4)–C(5) and C(4)–C(9) in the LUMO (Figure 4), thus, confirming the electronic back-donation from the metal fragment to curcumin.

The atomic orbitals of the C(4), C(5), and C(9) atoms participate in both HOMO and LUMO molecular orbitals in Cur-Enol. The C(4)–C(5) and C(4)–C(9) bonds in the HOMO are binding, but the C(4)–C(9) bond in the LUMO is anti-binding (Figure 4). Due to electronic transfer through LUMO back-donation or HOMO electronic donation, the bonds C(4)–C(5) and C(4)–C(9) lengthen. This conclusion is insufficient to account for the observed C(4)–C(9) bond shortening.

### 3.4. Energy Analysis of Cur-M Complexes (M = Ni, Cu, and Mg)

The Ziegler–Rauk energy decomposition method [34] was used to assess the relative importance of the covalent and ionic contributions to the binding energy (BDE). This formal fragmentation treated the curcumin ligand as an organic moiety (L1) and the remaining moiety as an organometallic part (L2). The binding energy BDE was divided into three components using the “single point” calculation with PW91/TZP and the ZORA approximation: the Pauli energy, orbital energy, and electrostatic energy (shown in the Table 4).

According to the Ziegler–Rauk method, the degree of covalency in the metal fragment and curcumin bond agrees with Hirshfeld charges. The Cur-Mg boundary orbital nodal characteristics reflect the poor covalency (8%).

#### 3.4.1. ETS-NOCV Analysis

The ETS-NOCV fragmentation analysis was carried out in the same way as before. The total interaction energies of the Cur-M complexes are similar. The interaction may be thought of as an ionic bond. Although the E_elec_ and E_orb_ values are close, the stabilizing electrostatic interaction is more significant than the orbital interaction. The results of energy decomposition obtained according to this method are shown in Table 5.

Compared to the other investigated systems, the cur-Mg complex has a lower covalent binding (9%) and a higher electrostatic term (91%). Consequently, the ETS-NOCV approach produces decomposition energies that increase in the same way as the fragmentation of Ziegler–Rauk (Table 4).

**Figure 5:**
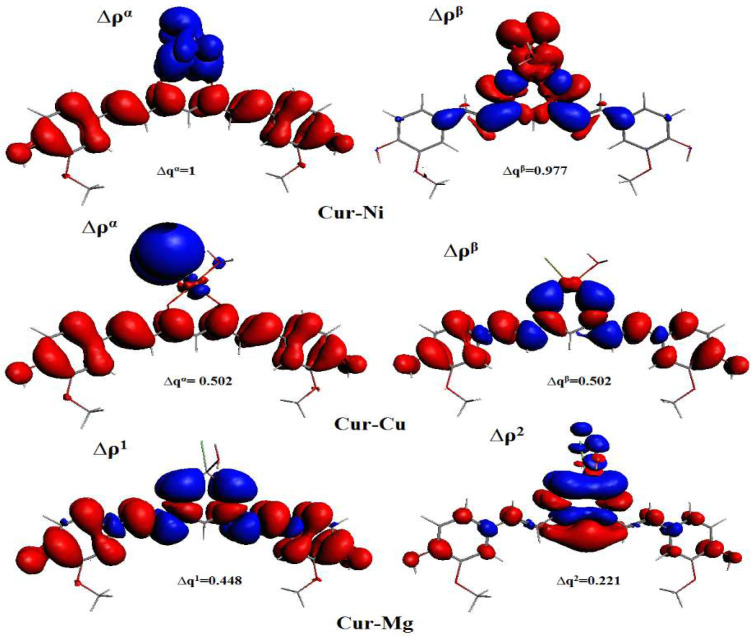
Natural orbitals for chemical valence (NOCV) pairs contours in cur-M (M = Ni, Cu, and Mg). Isovalue = 0.001.

Figure 5 shows the natural orbitals that have the most significant impact on the metal-curcumin bond, as well as the deformation density. Red represents areas of electronic density depletion, whereas blue represents areas of electronic density accumulation. From the red zones to the blue zones, the electrical density shifts accordingly. The orbital diagram depicts a back-donation of the metal fragment to curcumin (Figure 5).

#### 3.4.2. QTAIM Topological Analysis

The QTAIM parameters were obtained by a single point calculation on the geometries optimized with GGA: PW91/TZP. The most important point in this analysis is the presence of an additional critical point between the central portion (β-diketone), the hydroxyl groups on phenyl rings, and the oxygen on methoxy groups.

QTAIM descriptors were computed at critical points for each complex, and their values are cited in Table 6. The electron density and its Laplacian for intramolecular hydrogen bonds were included in the study. Curcumin and its complexes have hydrogen bonding properties, including as donor and acceptor.

**Figure 6:**
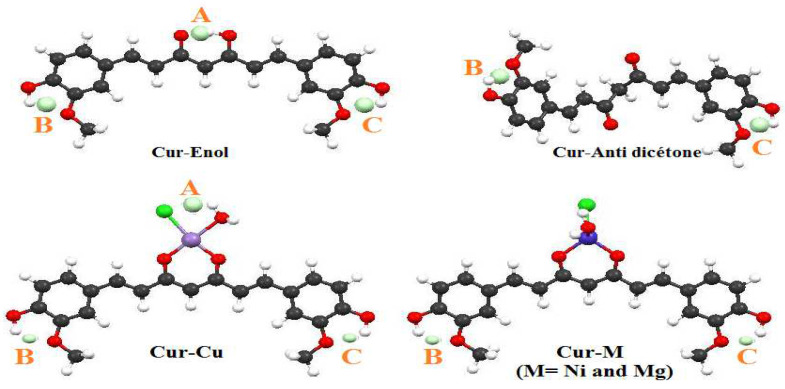
Representation of the critical points of the Cur-Enol, Cur-Anti diketone, and the Cur-M complexes (M = Ni, Cu, and Mg). (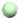: critical point).

Intramolecular hydrogen bonding was based on the electronic densities and their Laplacian values (Table 6 and Figure 6).

The chelation of curcumin with the metal pieces created two conformations: pseudo-tetrahedral for Cur-Ni and Cur-Mg and pseudo-plane square for Cur-Cu. Hydrogen bonding (Cl…HOH) is enabled by the copper square-planar coordination, which is preferred by Cu(II) compounds in general (Table 6 and Figure 6).

#### 3.4.3. Effect of Curcumin Complexation on Its Antioxidant Character

The BDE method was used to determine the phenolic hydrogen atoms dissociation energies to better understand the influence of curcumin complexation on its antioxidant properties. The ionization potentials (IP) of the Cur-Ni, Cur-Cu, and Cur-Mg complexes are indicated in Table 7.

In the case of the antioxidant action with hydrogen transfer mechanism, Cur-Enol remained the most active species. The Cur-Mg complex exhibited an IP = 115.45 Kcal mol^−1^ value for the electron transfer antioxidant mechanism. The Cur-Mg shows the best antioxidant activity explained by its values of HOMO energies (as best electron donor). The BDE and PI energies are lower than those of Cur-Ni and Cur-Cu.

#### 3.4.4. The Solubility of Curcumin and Its Complexes

For an antioxidant compound, high solubility is one of the most important physicochemical and biopharmaceutical criteria for use as an active agent in a drug or food supplement [45].

**Figure 7:**
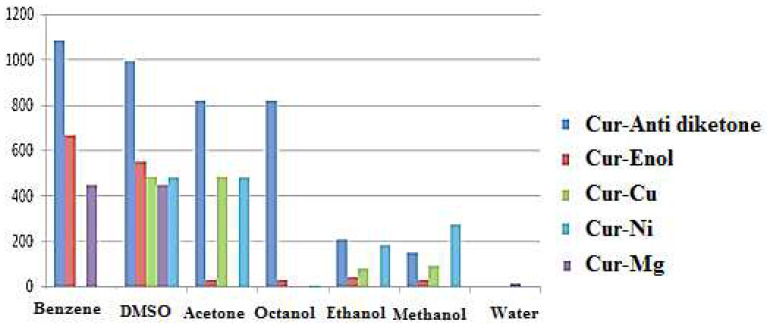
Solubility (g/L) of curcumin and its complexes Cur-M (M = Ni, Cu, and Mg).

Curcumin is a lipophilic amphiphilic molecule with therapeutic properties [12]. Its solubility in water must be improved to boost curcumin’s bioavailability [46]. The aromatic groups in curcumin structure provide hydrophobicity, which makes it poorly soluble in water, while acetone, ethanol, and benzene are suitable solvents. The most widely used solvent is still DMSO [47]. The solubility of curcumin tautomers and its complexes (Cur-Enol, Cur-Anti-diketone, and Cur-Ni, Cur-Cu, and Cur-Mg) were investigated (Table 8 and Figure 7).

In all solvents except water, Cur-Anti-diketone is the most soluble compound. Cur-Enol dissolves well only in benzene and DMSO but not in other solvents. The Cur-Mg complex is weakly soluble in water (14 g/L) but considerably superior to the other compounds, which are insoluble in water. Consequently, complexation can increase the water solubility of curcumin. In terms of solvents, only DMSO can dissolve all of the molecules (curcumin and its complexes).

## 4. Conclusions

DFT quantum calculations highlighted interesting properties of curcumin and its complexes, i.e., Cur-M (M = Ni, Cu, and Mg). The enolic form of curcumin (Cur-Enol) was found to be energetically more stable and biologically more active compared with its Cur-Anti-diketone tautomer. Furthermore, the antioxidant properties of Cur-Enol calculated by both ETS-NOCV and BDE methods were found to be better compared with Cur-Anti-diketone and its complexes. A combined ETS with the NOCV approach was also conducted to study the nature of curcumin-metal (Cur-M) bonding, and the results showed that all Cur-M bonding had a primarily ionic character (Cur-Ni: 71%, Cur-Cu: 57%, and Cur-Mg: 91%). QTAIM results showed the existence of two hydrogen bonds for Cur-Enol and Cur-Cu, which explained their stability compared to Cur-Mg and Cur-Ni.

The TDDFT calculated absorption and emission transitions of curcumin gave peaks that were similar to the published experimental findings with some shifts. The first excitation had the highest probability and was of the HOMO-LUMO type ($\pi \to \pi^{*}$ electronic transition). Solvatochromism existed in both absorption and emission and was found to be weak. We concluded that the HOMO–LUMO transition in Cur-Enol in all solvents was the most present in the UV-vis region in absorption and emission.

The theoretical descriptors BDE and PI, for which the antioxidant activity showed that the compound Cur-Mg was the best antioxidant, presented the values of BDE and PI as the lowest on two sites with (143.36–143.35) and 115.45 kcal/mol, respectively. This result was confirmed by the highest value of the HOMO structure.

Solubility calculations demonstrated that both Cur-Enol and Cur-Anti-diketone tautomers were soluble in DMSO but insoluble in water. Only Cur-Mg had a low water solubility (14 g/L), indicating that complexation promoted curcumin water solubilization and improved the therapeutic effects.

## Figures and Tables

**Table 1:** Values of ETS-NOCV, BDE, IP, and EA in (Kcal/mol) of Cur-Enol and Cur-Anti-diketone in DMSO.

	Position	ETS-NOCV	BDE	IP	ΔE_excit_
Cur-Enol	β	254.08	136.12	125.10	123.13
	γ	253.66	135.73		
	α	285.79	161.85		
Cur-Anti diketone	β	256.18	136.80	131.64	132.99
	γ	256.30	136.84		

**Table 2:** Comparison of the maximum absorption/emission, f, and ∆λ of curcumin in some solutions. Experimental data counterparts are reported between brackets [34].

Cur-Enol	Absorbance (nm)		Emission (nm)		Stokes’s Shift
	$\lambda_{Théo}^{Abs}$	f	$\lambda_{Théo}^{Em}$	f	∆λ
DMSO	454.18 (434)	1.7086	529.05 (518)	2.0297	75 (84)
Water	451.67 (414)	1.6829	530.19 (537)	2.0338	78 (123)
Methanol	451.08 (422)	1.6819	527.73 (534)	2.0250	77 (112)
Ethanol	451.96 (422)	1.6925	526.36 (526)	2.0199	74 (104)
THF	451.24 (422)	1.7066	514.11 (477)	1.9723	63 (55)
Acetonitrile	451.68 (418)	1.6869	528.10 (507)	2.0264	76 (89)
Cyclohexane	445.46 (408)	1.7128	478.81 (437)	1.8010	34 (29)

**Table 3:** Angles (°), Dihedral angle (°), and bond lengths (Å) for the studied complexes curcumin-Metal (Cur-M) where (M = Ni, Mg, and Cu), and length of the bonds C(4)–C(5) and C(4)–C(9) (Å), for curcumin and its complexes calculated at the PW91/TZP level.

	Pseudo-Tetrahedral		Pseudo-Square		
	Cur-Ni	Cur-Mg	Cur-Cu	Cur-Enol	Cur-Anti Diketone
Angles (°)					
Cl–M–O(3)	92	99	82	/	/
Cl–M–O(2)	124	123	95	/	/
O(2)–M–O(1)	96	95	96	/	/
O(1)–M–O(3)	103	105	87	/	/
Dihedral angle (°)					
Cl–O(3)–O(1)–O(2)	281	282	356	/	/
Bond length (Å)					
M–O(1)	1.939	1.964	1.925	/	/
M–O(2)	1.925	1.965	1.921	/	/
M–O(3)	2.095	2.089	2.127	/	/
M–Cl	2.247	2.320	2.313	/	/
C(4)–C(5)	1.407	1.414	1.406	1.371	1.521
C(4)–C(9)	1.411	1.415	1.408	1.430	1.521

**Table 4:** Decomposition in orbital terms E_Pauli_, E_elec,_ and E_Orb_ (eV) and Charge Metal of Cur-M complexes (where M = Ni, Cu, and Mg).

	E_Pauli_	E_elec_	E_Orb_	%Cov	%Ion	Charge Metal
Cur-Ni	5.5076	−7.4296	−10.4167	16	84	0.446
Cur-Cu	6.0406	−13.6989	−9.6546	44	56	0.436
Cur-Mg	2.6042	−3.3271	−8.6136	08	92	0.488

**Table 5:** The results of ETS analysis of cur-M complexes (M = Ni, Cu, and Mg). Values are expressed in kcal/mol.

	E_Pauli_	E_Orb_ (%)	E_elec_ (%)	E_int_
Cur-Ni	130.38	−236.11 (29%)	−264.62 (71%)	−370.35
Cur-Cu	138.37	−283.76 (43%)	−195.43 (57%)	−340.82
Cur-Mg	59.89	−79.92 (09%)	−198.31 (91%)	−218.34

**Table 6:** Topological properties of the critical point (A, B, and C) for hydrogen bonds (in atomic units) of curcumin and its complexes Cur-M (M = Ni, Cu, and Mg).

	ρ(r)	$\nabla^{2}\rho \left(r\right)$	|V|/G	H(r)
		Cur-Enol		
B	0.0220	0.1081	0.8652	0.0032
C	0.0220	0.1082	0.8651	0.0032
A	0.1066	0.0816	1.7163	−0.0515
		Cur-Anti diketone		
B	0.0220	0.1082	0.8654	0.0032
C	0.0220	0.1082	0.8657	0.0032
		Cur-Ni		
B	0.0221	0.1079	0.8669	0.0032
C	0.0217	0.1084	0.8620	0.0032
		Cur-Cu		
B	0.0220	0.1081	0.8655	0.0032
C	0.0219	0.1083	0.8645	0.0032
A	0.0240	0.0773	0.9529	0.0009
		Cur-Mg		
B	0.0220	0.1077	0.8658	0.0032
C	0.0219	0.1080	0.8648	0.0032

**Table 7:** Values of BDE, IP (in kcal mol^−1^), and HOMO of curcumin active positions (A, B, and C) and its complexes Cur-M (where M = Ni, Cu, and Mg).

	Positions	BDE	IP	HOMO
Cur-Enol	B	136.12	125.10	−5.184
	C	135.73		
	A	161.85		
Cur-Anti-diketone	B	136.84	131.64	−5.392
	C	136.80		
Cur-Ni	B	149.60	118.37	−5.164
	C	149.20		
Cur-Cu	B	143.60	147.35	−5.208
	C	143.58		
Cur-Mg	B	143.36	115.45	−5.073
	C	143.35		

**Table 8:** Solubility (g/L) of curcumin and its complexes in different solvents.

	Benzene	DMSO	Acetone	Octanol	Ethanol	Methanol	Water
Cur-Anti diketone	1083.23	991.50	821.19	821.19	208.44	146.99	0.00112
Cur-Enol	664.76	549.83	29.97	29.97	43.41	29.85	0.00011
Cur-Ni	0.0090	479.54	479.54	4.12	183.38	274.28	0.0043
Cur-Cu	0.00020	484.39	484.39	1.22	77.58	90.86	0.0009
Cur-Mg	445.15	445.15	1.64	9.04E5	0.0049	0.0026	14.44

## Data Availability

Not applicable.
